# Quality assessment of paediatric education and research in southwest China: A cross-sectional study

**DOI:** 10.1371/journal.pone.0301708

**Published:** 2024-04-05

**Authors:** Jing Gao, Haining Zheng, Tingting Wu, Jing Zhu

**Affiliations:** 1 Children’s Hospital of Chongqing Medical University, Chongqing, China; 2 Chongqing Collaborative Innovation Center for Functional Food, Chongqing University of Education, Chongqing, China; 3 Department of Food and Nutrition, College of Medical and Life Sciences, Silla University, Busan, South Korea; 4 Ministry of Education Key Laboratory of Child Development and Disorders, Pediatric Research Institute, Children’s Hospital of Chongqing Medical University, Chongqing, China; 5 China International Science and Technology Cooperation Base of Child Development and Critical Disorders, Chongqing, China; 6 Chongqing Key Laboratory of Pediatrics, Chongqing, China; King Abdulaziz University Faculty of Medicine, SAUDI ARABIA

## Abstract

Currently, there is a limited analysis of medical education research both domestically and internationally. To enhance, improve the quality of medical education, this study conducted a quantitative analysis of teaching project data from an affiliated hospital during the period 2016–2022. The results indicated that a total of 133 teaching projects were initiated during this period, with an average age of project leaders being 42.73±6.45 years. Regarding professional ranks and titles, municipal-level project leaders had a high concentration of seniors (48.15%), while at the university-level, most project leaders held the title of deputy seniors (58.82%). At the university-level, project leaders were mainly distributed between deputy senior titles (37.08%) and intermediate titles (38.20%). In terms of research content, nearly half of the studies (46.62%) focused on teaching methods and models. Further regression analysis revealed that professional ranks and titles were an independent factor influencing the project level (P<0. 05). These findings suggest the need for improvement in medical education research, including addressing the uneven distribution of research topics, enhancing the research capacity of junior and mid-career medical education teachers, and improving the dissemination of research results.

## Introduction

### Medical education research and its importance

In recent years, China has witnessed the rapid development in its medical education system, which has been actively explored and implemented to deepen educational reform and improve the quality of education. The outbreak of COVID-19 in 2019 and the changing global landscape have highlighted the importance of healthcare. Consequently, there is an urgent need to expedite the transition from a disease-centered to a health-centered approach in talent training, while incorporating fresh perspectives and new evidence into medical education research. Findings from medical education research can influence the learning and teaching decisions of medical students and researchers, providing new facts, concepts, ideas, and addressing emerging issues [1]. Therefore, medical education research holds significant implications for enhancing medical teaching and learning. In September 2019, the Ministry of Education of the People’s Republic of China issued the "Opinions on Deepening the Reform of Undergraduate Education and Teaching for Overall Improvement in the Quality of Talent Cultivation," which proposed new and higher requirements for medical education and theoretical research in China [2]. These guidelines offer valuable insights for medical educators, aiding them in guiding their teaching practices and engaging in educational research by identifying the direction and goals of medical education research.

### What is the role of the hospitals that are affiliated to universities?

The unique relationship between affiliated hospitals and medical universities is characterized by the distinctive features and principles of medical education. Affiliated hospitals bear the responsible of clinical teaching, scientific research, and talent training, serve as the primary site for clinical teaching and practice in higher medical institutions. They also serve as a platform for disseminating medical knowledge, medical humanities, and scientific research findings. During clinical practice, faculty members impart the principles of evidence-based medicine (EBM) through their clinical experience, enabling medical students to comprehend the scientific evaluation of clinical research evidence from the perspective of clinical epidemiology. This equips them with the ability to properly understand and apply EBM, fostering scientific thinking and innovative skills [3]. For instance, bedside teaching facilitates an immersive clinical environment, exposes students to clinical culture, and imparts specialized clinical knowledge [4]. Affiliated hospitals attach great importance to teaching, and the implementation of teaching and research projects serves as an effective approach to transform teaching concepts, promote educational reform, and enhance the quality of medical personnel training. By identifying and analyzing issues in clinical teaching practice and converting them into medical education research projects, the quality of medical education can be improved. Therefore, updating teaching concepts in affiliated hospitals and keeping pace with the changing times are vital aspects of ensuring the quality of medical education. The development of affiliated hospitals is closely intertwined with the reform of medical education both domestically and internationally. However, the persistent phenomenon of blindly following research trends persists, with recurring themes emerging over the years. Researchers often overlook their own context and research topics, lacking a deep understanding of these trends and failing to consider their relevance to their research goals. Consequently, their adoption and application of these hotspots become mechanical and merely a matter of following the crowd. The selection of research topics around hotspots exhibits significant fluctuations, making it challenging to conduct in-depth investigations into long-term or relatively stable research directions. The prevalence of low-quality teaching research projects has become commonplace [5]. An analysis of teaching research projects in university-affiliated hospitals in east China reveals that the level of projects is mainly dominated by university-level and college-level projects, with teaching methods and models account for 80% of the research content. There is a lack of cross-sectional studies, and the scope of teaching project research is not sufficiently broad [6]. Therefore, researchers have called for high-quality medical education research projects and outcomes to address these issues [7, 8]. On the one hand, medical education research should be based on the current status and trends of domestic and international medical higher education, closely follow the issues of medical education development and reform. It should address the new problems and challenges of contemporary medical education and actively tackle important and difficult issues, thereby enhancing the research and teaching reform capabilities of medical education [9, 10]. On the other hand, each study should systematically build upon previous research and incorporate future studies to have a positive impact on clinical practice [11].

Currently, there is a limited analysis of medical education and teaching research conducted in university-affiliated hospitals, both domestically and internationally. In this article, we conducted an in-depth analysis of education and teaching research projects in a leading university-affiliated children’s hospital in southwest China. The aim of this analysis was to enhance the quality of medical education research. The analysis spanned from 2016 to 2022 and was conducted in accordance with China’s undergraduate medical education standards. This study aimed to provide a quantitative basis for future resource allocation towards education, teaching reform, and research, as well as to further enhance the quality of talent development.

## Methods

This research is derived from education reform project is exempt from Ethics Committee of the Children’s Hospital of Chongqing Medical University. Written informed consent was obtained from all participants in this study, and all data from the teaching reform project were anonymized and subsequently evaluated and analyzed by the Office of Academic Affairs.

The data for this study were collected from the education and teaching research projects conducted at the Children’s Hospital of Chongqing Medical University between 2016 and 2022. A total of 133 projects were identified and categorized into three groups: municipal, university, and college. The municipal education and teaching research projects included contributions from the Chongqing Education Commission, the Chongqing Institute of Education Sciences, the Chongqing Higher Education Society, and the Chinese Medical Association. The university education research projects involved Chongqing Medical University, and the college education and teaching research projects involved the Children’s Hospital of Chongqing Medical University.

The research topics mainly focused on ten aspects of teaching, including teaching methods and modes, curriculum construction, teaching platform and resource construction, experiments, practical teaching and effects, quality assurance system, medical humanities education and professional quality, ideological and political education, modern educational philosophy and practice, talent training system and faculty development.

Data analysis was performed using SPSS version 21.0 and Excel. Count data were expressed as percentages (%), while measurement data were expressed as mean ± standard deviation (X ± S). Factors influencing the initiation of teaching projects were analyzed using multivariate ordered logistic regression, with a significance level set at α = 0.05 (two-tailed). The detailed results are shown in Table 1.

**Table 1:** Factors influencing approval of teaching projects, 2016–2022.

Variable	Assignment
Project level	1 = College-level
	2 = University-level
	3 = Municipal-level
Age	1 = 30–39 2 = 40–49 3 = 50–59
Professional ranks and titles	1 = Junior
	2 = Intermediate
	3 = Deputy senior
	4 = Senior
Academic degree	1 = Bachelor 2 = Master 3 = Doctor
Research content	1 = Teaching method and modes
	2 = Curriculum construction
	3 = Teaching platform and resource construction
	4 = Experiments, practical teaching and effects
	5 = Quality assurance system
	6 = Medical humanities education and professional quality
	7 = Ideological and political education
	8 = Modern educational philosophy and practice
	9 = Talent training system
	10 = Faculty development

## Results

**Fig 1:**
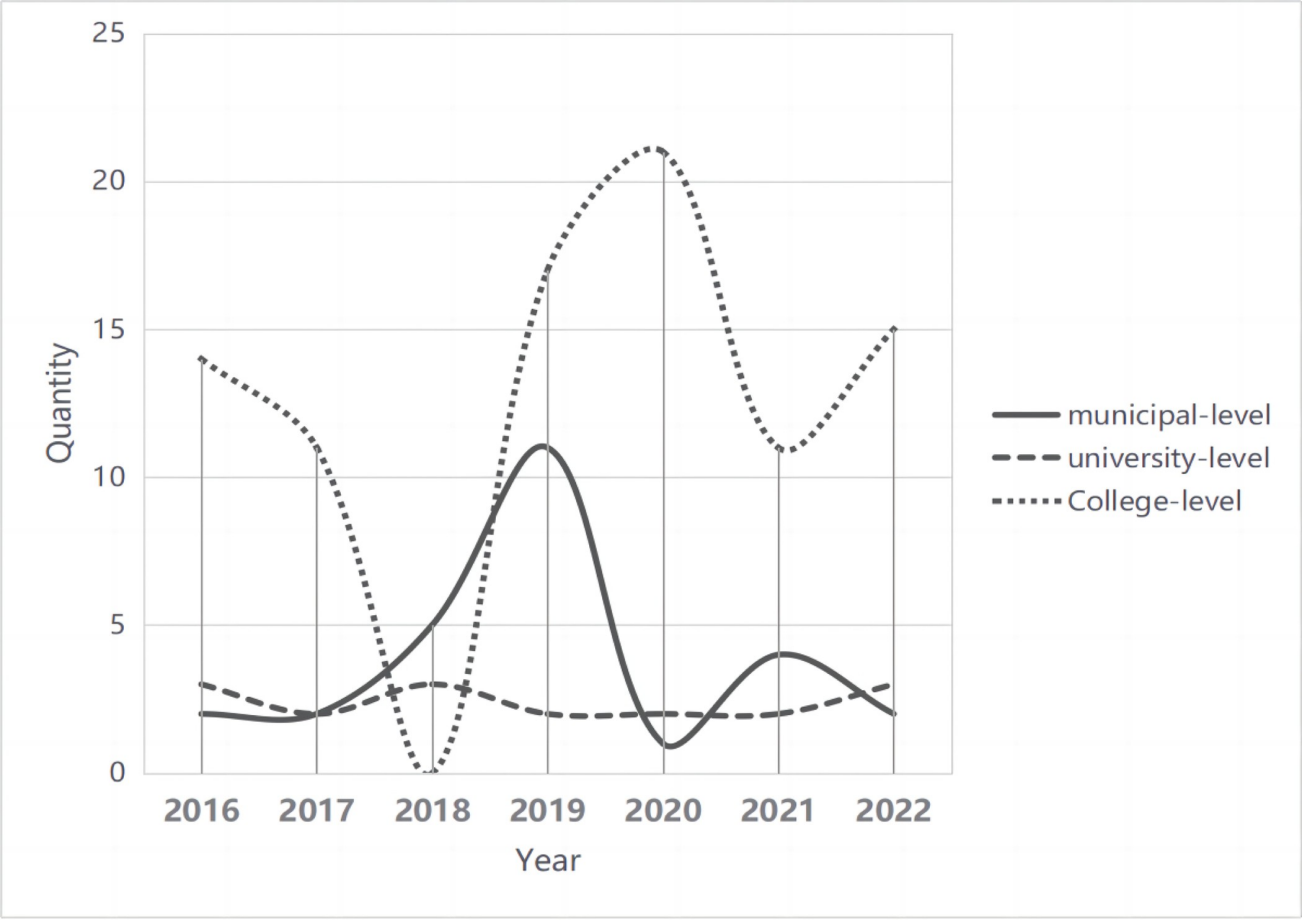
Distribution of research content of education and training topics 2016–2022.

From 2016 to 2022, a total of 133 teaching and teaching research projects were established in the affiliated hospitals. These projects include 27 projects at the municipal-level, 17 projects at the university-level, and 89 projects at the college-level. However, no teaching research projects were identified at the national level. The majority of education and training projects are at the college-level. The distribution of the different project types on an annual basis is shown in Fig 1.

The results show that the research topics of education and teaching projects cover various areas: teaching methods and modes (46.62%), curriculum construction (15.79%), teaching platform and resource construction (6.77%), experiment, practice teaching and effect (10.53%), quality assurance system (6.77%), medical humanities education and professional quality (5.26%), ideological and political education (3.01%), modern education concepts and practice (2.26%), talent training system (2.26%), and teacher training (0.75%). The research theme of education and teaching research projects primarily focuses on teaching methods and modes, although the research content of education and teaching research projects encompasses ten aspects (Table 2).

**Table 2:** Information form for those involved in teaching and learning projects 2016–2022.

Parameter	Total	Municipal-level	University-level	College-level	P
Age					
30–39	50(37.59)	9(33.33)	6(35.29)	35(39.33)	0.061
40–49	66(49.63)	10(37.04)	9(52.94)	47(52.81)	
50–59	17(12.78)	8(29.63)	2(11.77)	7(7.86)	
Professional ranks and titles					
Junior	6(4.51)	2(7.41)	1(5.88)	3(3.37)	0.021
Intermediate	45(33.83)	8(29.63)	3(17.65)	34(38.2)	
Deputy senior	47(35.34)	4(14.81)	10(58.82)	33(37.08)	
Senior	35(26.32)	13(48.15)	3(17.65)	19(21.35)	
Academic degree					
Bachelor	8(6.02)	0(0.00)	0(0.00)	8(8.99)	0.235
Master	54(40.60)	10(37.04)	6(35.29)	38(42.70)	
Doctor	71(53.38)	17(62.96)	11(64.71)	43(48.31)	
Research content					
Teaching method and modes	62(46.62)	12(44.44)	5(29.42)	45(50.56)	0.015
Curriculum construction	21(15.79)	1(3.70)	4(23.53)	16(17.97)	
Experiments, practical teaching and effects	14(10.53)	2(7.41)	1(5.88)	11(12.36)	
Teaching platform and resource construction	9(6.77)	5(18.52)	1(5.88)	3(3.37)	
Quality assurance system	9(6.77)	2(7.41)	1(5.88)	6(6.74)	
Medical humanities education and professional quality	7(5.26)	4(14.82)	1(5.88)	2(2.25)	
Ideological and political education	4(3.01)	0(0.00)	2(11.77)	2(2.25)	
Talent training system	3(2.25)	0(0.00)	1(5.88)	2(2.25)	
Modern educational philosophy and practice	3(2.25)	1(3.70)	0(0.00)	2(2.25)	
Faculty development	1(0.75)	0(0.00)	1(5.88)	0(0.00)	
Total	133(100.00)	27(100.00)	17(100.00)	89(100.00)	

The project leader has full responsibility for the management and execution of the research project. In terms of age, the average age of individuals involved in education and training projects is 42.73 ± 6.45 years. The age of project leaders engaged in education and training projects at the municipal, university and college level is 44.93 ± 7.16, 42.53 ± 6.53 and 42.10 ± 6.14 years, respectively. In particular, project leaders at the university and college level tend to be younger than those at the municipal level. Regarding titles and degrees, the majority of project managers at the municipal level hold a senior title (48.15%), with 37.04% holding a master’s degree and 62.96% a doctorate. At the university level, the majority of project managers hold the title of deputy senior (58.82%), with 35.29% holding a master’s degree and 64.71% a doctorate. At the college level, project managers are predominantly distributed among deputy senior (37.08%) and intermediate (38.20%), with 42.70% holding a master’s degree and 48.31% holding a Ph.D (Table 2).

Multivariate analysis was conducted with the level of project approval (1 = college level, 2 = university level, 3 = Municipal level) as the dependent variable and the significant factors identified in the univariate analysis as the independent variables. The results revealed that professional ranks and titles can influence the establishment level of teaching projects (p<0.05). The odds ratios (OR) and 95% confidence intervals (CI) for deputy senior and intermediate, compared to senior, were 2.987 (1.031, 8.655) and 3.975 (1.374, 11.501) respectively. These findings suggest that the level of project establishment for subject leaders with deputy senior and intermediate ranks is significantly lower than that for subject leaders with senior rank (Table 3).

**Table 3:** Results of multivariate ordered logistic regression analysis of the factors influencing the approval of teaching projects in 2016–2022.

Variable	Estimate	Wald	P	OR	95% CI	
					Lower Bound	Upper Bound
**R variable Y**						
Municipal-level	-0.421	0.264	0.608	0.657	0.132	3.271
University-level	0.359	0.193	0.661	1.431	0.288	7.100
**Explanatory variable X**						
Professional ranks and titles						
Junior	0.983	0.862	0.353	2.673	0.335	21.305
Intermediate	1.380	6.483	0.011	3.975	1.374	11.501
Deputy senior	1.094	4.065	0.044	2.987	1.031	8.655
Research content						
Teaching method and modes	0.329	0.184	0.668	1.389	0.310	6.229
Teaching platform and resource construction	-1.241	1.51	0.219	0.289	0.040	2.092
Curriculum construction	1.252	1.819	0.177	3.497	0.567	21.561
Talent training system	0.122	0.007	0.933	1.130	0.065	19.673
Faculty development	-1.125	0.315	0.575	0.325	0.006	16.499
Experiments, practical teaching and effects	0.392	0.167	0.683	1.480	0.225	9.730
Ideological and political education	-0.418	0.115	0.734	0.659	0.059	7.328
Modern educational philosophy and practice	0.094	0.005	0.946	1.099	0.071	17.067
Medical humanities education and professional quality	-1.461	1.795	0.180	0.232	0.027	1.967

## Discussion

### The overall quality of the project requires improvement

According to relevant studies, high-quality medical education projects applied to the teaching environment [12] have been shown to provide guidance for both theoretical and practical teaching [13, 14]. In this review, a total of 133 projects approved by the institute between 2016 and 2022 were examined. Although the proportion of community-level projects is small, they can, to some extent, represent the current trends and frontiers of educational research in terms of national educational theory innovation and educational practice development. However, it is necessary to improve the overall quality of project application. Several possible explanations for these are as follows: Firstly, the clinical workload is cumbersome, which limits the ability to carry out significant research projects. Secondly, there is insufficient capacity for conducting educational research, and there is a lack of systematic educational research designs. Thirdly, compared to scientific research, teaching research lacks incentives, making it challenging to motivate clinicians and researchers.

To address these issues, several suggestions can be considered. Firstly, it is important to strengthen the quality of medical education research. This can be achieved by strengthening the hierarchical review process and considering expert opinions for projects that have not been approved. Secondly, there should be an emphasis on interpreting and training in education and teaching policies both domestically and internationally. This will help guide the direction of the project applications and ensure strict enforcement of mid-term inspections and final acceptance of the subjects that do not meet the expected results. Additionally, project approvals for multiple applications that do not meet the required standards should be canceled. Encouraging cooperation among institutions to conduct research, improving the teaching reward mechanism, and implementing top-level design are specific measures to ensure high teaching quality [15, 16]. Furthermore, the program of practical research should be guided by theoretical research in medical education. The post-competence and ability training of medical talents should serve as a guide for clinical teachers to conduct forward-looking theoretical research in medical education.

### The content of the research is unevenly distributed

Although education and teaching research projects cover a wide range of topics, these research themes mainly focus on teaching methods and modes, indicating an imbalance and centralization in the distribution of research content. Many similar research topics have been studied repeatedly over the years, with some blindly following these trends. For example, keywords such as ’problem-based learning (PBL)’, ’team-based learning (TBL)’, ’case study-based learning (CBL)’ and ’flipped the classroom’ appear frequently, suggesting a continuation of previous research. According to Chen MJ et al, TBL is more effective than lecture-based learning (LBL) in improving students’ knowledge, attitudes, and skills in theoretical teaching [7]. Additionally, the team-based CBL (TB-CBL), a new case-based teaching method, seems to yield similar learning outcomes and higher student satisfaction compared to small-group CBL [17]. Relevant research indicates that teachers’ content knowledge plays a crucial role in motivating students’ interests and hobbies [18]. Therefore, it is of utmost importance to compare the effectiveness of different teaching methods based on the form and needs of the students [19]. The repeated occurrence of topics with similar research themes over many years can be explained by several factors. Firstly, the guidance of national policies and the requirements for professional titles place emphasis on generating relevant teaching achievements, including teaching research and papers. This increases teachers’ enthusiasm to declare projects, resulting in repeated topic selection, insufficient innovation, and an overemphasis on researching teaching methods and models. Secondly, project approval processes may not adequately consider the current situation and direction of higher education research [9].

Looking back at the early development of medical education in China, it was predominantly characterized by a traditional education model. Medical students heavily relied on rote learning to pass examinations, resulting in a shallow understanding of "how to do it" without delving into the underlying reasons or "why they did it". Consequently, the teaching model became overly homogeneous, lacking specialization, diversity, and practical application, with students lacking the ability to independently explore and experiment. Recognizing these limitations, medical researchers in China have explored various "student-centred" teaching models, which have shown some success.

Currently, medical education in China is undergoing a new phase of development that centers around health and encourages interdisciplinary cross-fertilization. In light of these new opportunities and needs, medical educators should engage in medical education research and critically reflect on how to reform the education and teaching system. However, it is observed that many medical educators remain focused on researching teaching models and methods, neglecting crucial aspects such as curriculum development, talent system construction, and teaching resources. Therefore, the selection of research topics becomes the crucial starting point for medical education research [20]. It is important to note that asking the right questions often holds more significance than merely addressing an issue [21].

The essence of topic selection lies in formulating questions that bridge the gap between the ’known’ and the ’unknown’ in teaching activities. Therefore, it is imperative to engage in thoughtful planning, promote effective integration of various disciplines and majors, avoid blind replication, prevent the "convergence" of repetitive research, and prioritize the development of innovative topics. Additionally, there should be a focus on strengthening the construction of talent training system and enhancing the quality of educational research [12, 22]. By addressing these considerations, the field of medical education in China can advance towards a more comprehensive and impactful approach that fosters independent thinking, creativity, and continuous improvement in the teaching and learning process.

### Improving the training, teaching, and research skills of junior and mid-career faculty

According to the results of the analysis, subject teachers with deputy senior and intermediate positions exhibit a significantly lower level of project establishment compared to subject teachers with senior positions. In China, young teachers have emerged as the primary driving force in higher education. They fulfill their specialized teaching responsibilities and contribute to the training of modern talent the country’s comprehensive development. Therefore, it is imperative to prioritize their training early on, both for their professional growth and the advancement of education [23]. Thematic research serves as an effective approach for the rapid development of young teachers. Engaging in thematic research facilitates the enhancement of teachers’ skills in "reading", "speaking" and "writeing". "Reading" involves extensive research conducted domestically and internationally, while "speaking" encompasses classroom practice. Lastly, "writing" focuses on refining teaching methodologies and conducting research to improve educational practices.

### The achievement and promotion are insufficient

Some of the teachers’ research accomplishments are disseminated through papers, while others take the form of research reports. These research outputs have been recognized and honored with China’s Higher Education Achievement Awards, reflecting their significant impact. China’s teaching achievements are evaluated every four years. Upon reviewing the list of China’s higher education achievements from the past decade, it becomes evident that there is a dearth of pediatric education-related accomplishments, which aligns with the findings reported by Meo et al [18]. Consequently, it is crucial to strengthen the analysis of education and teaching research projects and their corresponding achievements. This will serve two purposes: firstly, it will prevent the proliferation of low-quality and redundant research, thus enhancing the outcomes of medical research. Secondly, it will facilitate subsequent researchers in building upon and referencing previous studies, fostering longitudinal research coherence and propelling the advancement of education and teaching.

Furthermore, there exists a deficiency in the dissemination and promotion of teaching research results. It is imperative to not only focus on the generation of these research results but also on their effective translation into practical teaching methodologies. This concerted effort will foster the comprehensive advancement of education and teaching reforms. By actively engaging in diverse forms of education and teaching research and advocating for educational reforms, teachers contribute to the continual enhancement of both the overall standard of medical education and the level of research conducted in this field within the hospital setting.

## Limitations

This study did not encompass an analysis of cross-sectional studies within the realm of educational research. To ensure a more comprehensive understanding and statistical examination of research projects, it is advisable to explore additional perspectives. Therefore, it is recommended to incorporate a questionnaire survey targeting pediatric medical education researchers. This will enable an assessment of the quality of current pediatric medical education research from their distinctive viewpoints. Additionally, conducting comparative analyses with other research units may yield valuable insights and further enrich the findings of this study.

## Conclusion

The primary objective of medical research is to advance scientific knowledge, ultimately leading to enhanced disease treatment and prevention. This study found that the research topics of education and teaching projects primarily focused on teaching methods and modes, with relatively fewer studies on other aspects such as curriculum construction, teaching platform and resource construction, and quality assurance systems. This indicates that there is a potential for further development in these areas of medical education research. In addition, we found that the title and academic qualifications of project leaders had an impact on the quality and depth of educational research. Thus, expanding research domains, fostering interdisciplinary collaboration, enhancing professional development and teacher training, and promoting the dissemination of research findings can drive comprehensive growth in medical education research, elevate the quality of teaching, and ultimately advance scientific knowledge for more effective disease treatment and prevention.
